# Sex-Based Differences in Femoral Neck Circumference: A Cadaveric Morphometric Study

**DOI:** 10.7759/cureus.113891

**Published:** 2026-08-03

**Authors:** Zackary Sabetta, Steiv Shore, Kade Christensen, Lorenzo Petralia, Daniel Waszczuk, Owen Connell, Ava A Thielman, Sunny Patel, Michael Pollack, Sara Funk, Sumitra Miriyala

**Affiliations:** 1 Anatomy, A.T. Still University Kirksville College of Osteopathic Medicine, Kirksville, USA; 2 Anatomical Sciences, Arizona State University, Tempe, USA; 3 Medicine, A.T. Still University Kirksville College of Osteopathic Medicine, Kirksville, USA; 4 Ananomy, A.T. Still University Kirksville College of Osteopathic Medicine, Kirksville, USA

**Keywords:** cadaveric anatomy study, femoral neck morphometry, hip fracture risk, proximal femur geometry, sexual dimorphism bone

## Abstract

Introduction

Sex-based differences in femoral neck morphology may contribute to the disparate incidence of hip fractures between males and females. While prior studies have largely relied on imaging-based measurements, direct morphometric evaluation of femoral neck circumference in cadaveric specimens remains limited. This study examined sex-based differences in femoral neck morphology using a cadaveric model, with an emphasis on circumference as a novel direct measurement parameter.

Materials and methods

Twenty-five embalmed skeletally mature human cadavers (13 females, 12 males) were included. A total of 47 femora were extracted and measured, excluding femora with a prosthesis or hardware. Cadaver-level mean values were calculated by averaging bilateral measurements when available. Morphometric parameters included femoral neck circumference, neck-shaft angle, and femoral neck axis length. Independent-samples Welch t-tests were used to compare sex-based differences. Paired t-tests and Pearson correlation were performed to evaluate bilateral symmetry. Effect sizes were calculated using Hedges' g.

Results

Male cadavers demonstrated significantly greater mean femoral neck circumference compared to females (10.93 ± 0.64 cm vs. 9.45 ± 0.77 cm; Welch t(22.74) = 5.22, p < 0.001; Hedges' g = 2.00). No statistically significant sex-based differences were observed in neck-shaft angle (p = 0.47) or femoral neck axis length (p = 0.074). Strong bilateral symmetry was observed for femoral neck circumference (r = 0.88, p < 0.001).

Conclusion

Direct cadaveric measurement reveals clear sexual dimorphism in femoral neck circumference, whereas angular and linear dimensions show minimal sex-based differences. These findings suggest that circumferential cross-sectional geometry, rather than angular or linear morphology, may represent a meaningful axis of structural dimorphism in the proximal femur. Further biomechanical and imaging-based investigations incorporating femoral neck circumference are warranted.

## Introduction

The healthcare burden of hip fractures represents a significant and growing public health crisis, particularly in the elderly population. Hip fractures in older adults are associated with substantial morbidity, mortality, and healthcare costs, and recent epidemiological data demonstrate persistently high incidence and considerable first‑year post‑fracture mortality [1-3]. Global projections estimate that annual hip fracture cases will exceed six million by 2050, underscoring the urgent need for improved fracture risk stratification [4].

The majority of hip fractures occur at the femoral neck, a biomechanically vulnerable segment of the proximal femur subjected to varus, valgus, axial, and shear forces during daily activity and low-energy falls [5-7]. Structural parameters of the proximal femur - including femoral neck width, neck-shaft angle, and hip axis length - may influence fracture patterns independently of bone mineral density [5-7]. Prior meta-analytic evidence has demonstrated associations between proximal femoral geometry and femoral neck fracture risk, although findings vary across populations and measurement methods [8]. 

Sex-based differences in hip fractures are well documented: females account for approximately 70-75% of cases globally [1-3]. This disparity has been partially attributed to proximal femoral dimorphism and age-related skeletal changes, though the precise geometric contributors remain debated [8-13]. While females tend to demonstrate greater variability in neck-shaft angle and cortical thickness distribution, males with hip fractures experience higher post-fracture morbidity and mortality, suggesting that anatomical differences may influence outcomes beyond incidence alone [2,14,15].

Much of the existing literature on proximal femoral geometry has relied on dual-energy X-ray absorptiometry (DXA) or standard radiography, both of which are subject to projection distortion and resolution variability [11-13,16]. Direct cadaveric measurement offers a complementary approach that bypasses projection‑related and resolution‑dependent limitations inherent to radiographic and DXA‑based methods. However, comprehensive direct morphometric data stratified by sex, particularly regarding femoral neck circumference, remain scarce. This study addresses that gap by directly measuring femoral neck circumference - a parameter reflecting cross-sectional geometry and a determinant of the second moment of inertia - alongside neck-shaft angle and femoral neck axis length in a cadaveric cohort. These findings serve as a foundation for incorporating circumferential geometry into fracture risk models and orthopedic implant design considerations.

## Materials and methods

Study design and specimen selection

This single-center, cross-sectional morphometric study was conducted using embalmed human cadaveric specimens. Twenty-five skeletally mature cadavers were included (13 females, 12 males). The sex of each specimen was recorded from institutional donor records. Cadavers with evidence of severe osteoarthritis, prosthetic implants, or hardware at the hip were excluded. Cadaver height, weight, body mass index (BMI), age, and bone mineral density (BMD) were unavailable from institutional records and therefore could not be incorporated into the analysis. This represents a recognized limitation of the study and is addressed in the Discussion.

Bilateral femoral extraction was attempted for all specimens; however, complete bilateral measurements were not available in all cases due to extraction limitations. A total of 47 femora were successfully measured (22 right, 25 left). For bilateral correlation analysis, only cadavers with complete right and left circumference measurements were included (n = 22 paired cadavers; 44 femora). Measurements were performed by a single independent observer for consistency.

Surgical dissection and proximal femur excision

The femurs were internally rotated to tension the short external rotators. An incision was made at the tendinous attachment of the short external rotators to the greater trochanter. A second incision was made in the posterior hip capsule to provide optimal exposure of the femoral head and acetabulum. The femur was then internally rotated and abducted to achieve posterior dislocation of the hip and free the femoral head. A power drill with a cutting bit was applied to the mid-shaft incision mark to fully transect the femur; the specimen was then manually impacted with a mallet to reduce the risk of subtrochanteric fracture. The proximal femur was extracted and analyzed.

Morphometric measurements

**Figure 1:**
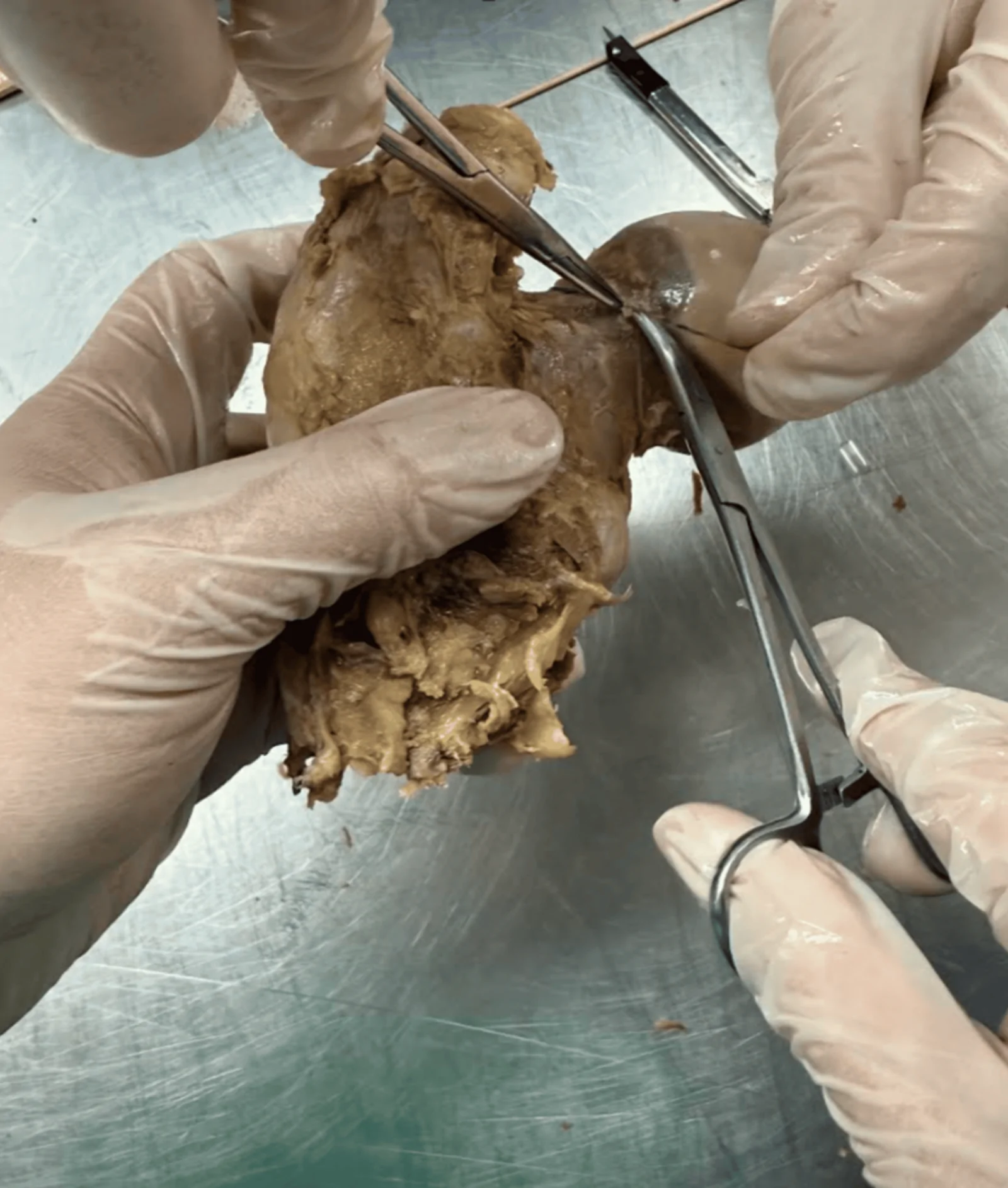
Circumference of femoral neck measurement

**Figure 2:**
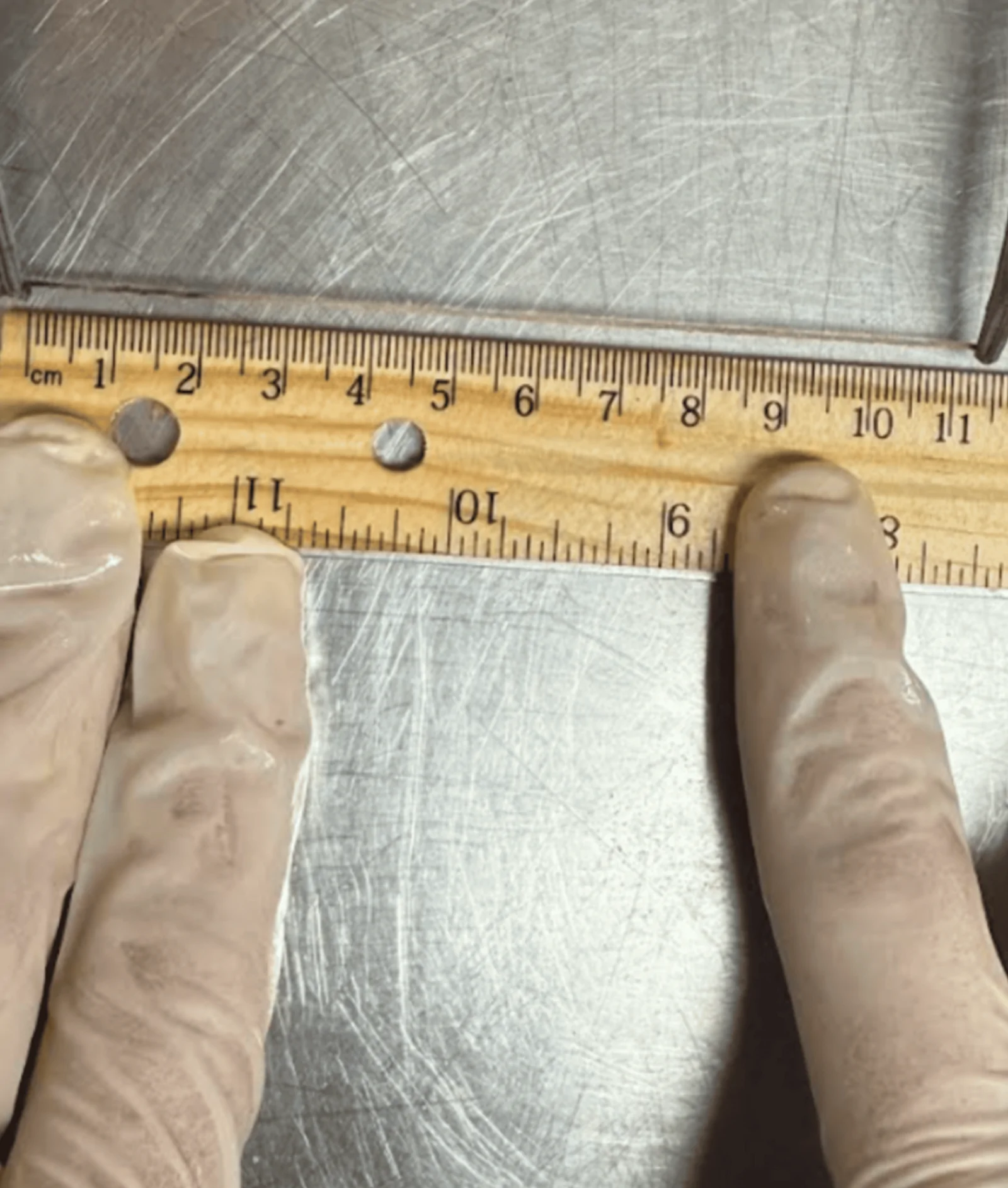
Measurement of string

Three measurements were obtained from each extracted proximal femur. Femoral neck circumference (cm) was obtained by wrapping an inextensible measuring filament around the anatomical narrowest point of the femoral neck (Figure 1). Needle drivers were used to clamp the filament at the point of overlap (Figure 2). The string was removed and its length measured against a millimeter ruler.

**Figure 3:**
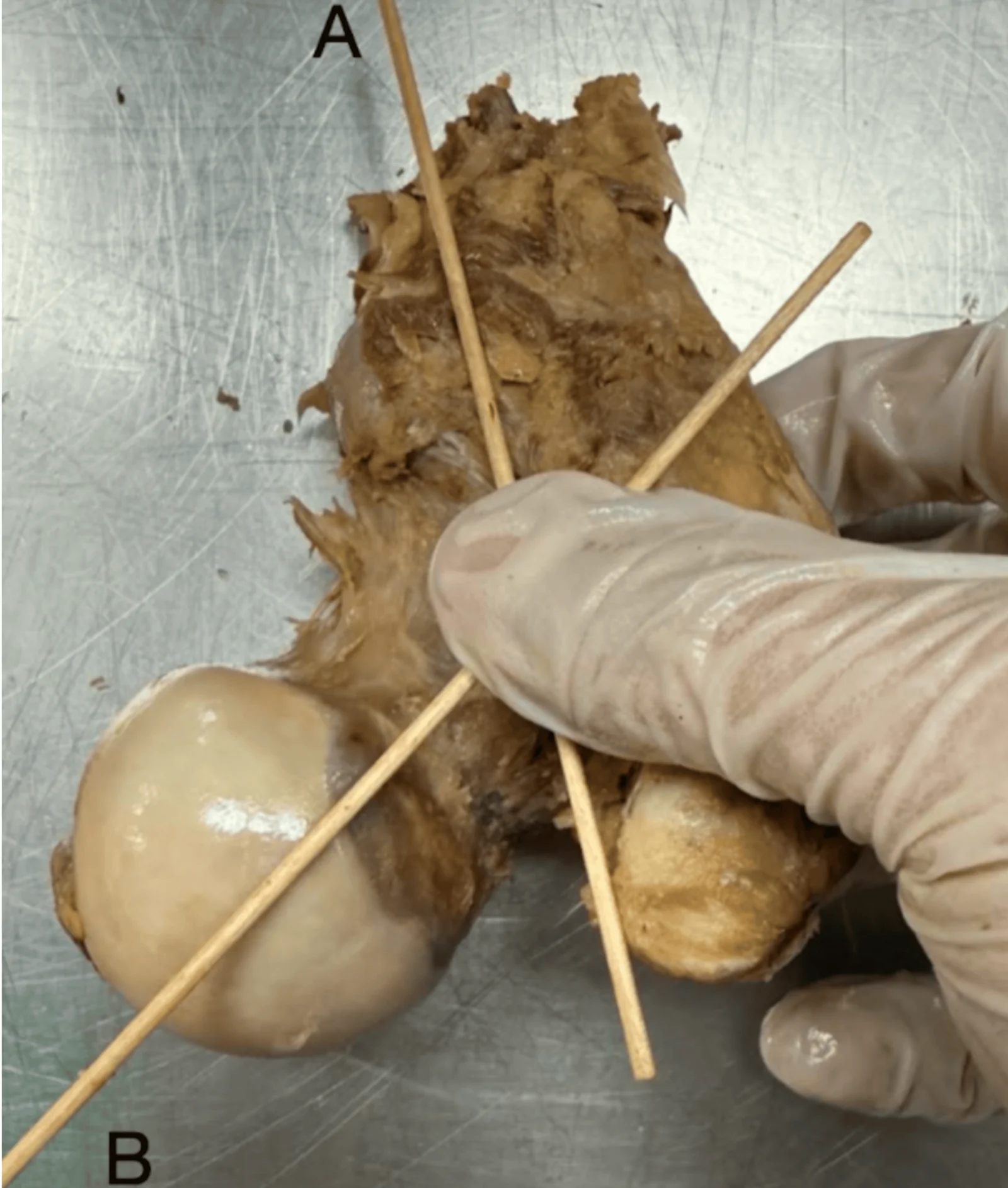
Angle measurement Stick A is aligned along the femoral shaft from the greater to the lesser trochanter, representing the long axis of the proximal femur. Stick B is positioned from the center of the femoral head to the midpoint of the intertrochanteric line, representing the femoral neck axis. The angle between sticks A and B is recorded as the neck‑shaft angle using a digital protractor application, with measurements verified against a physical protractor.

**Figure 4:**
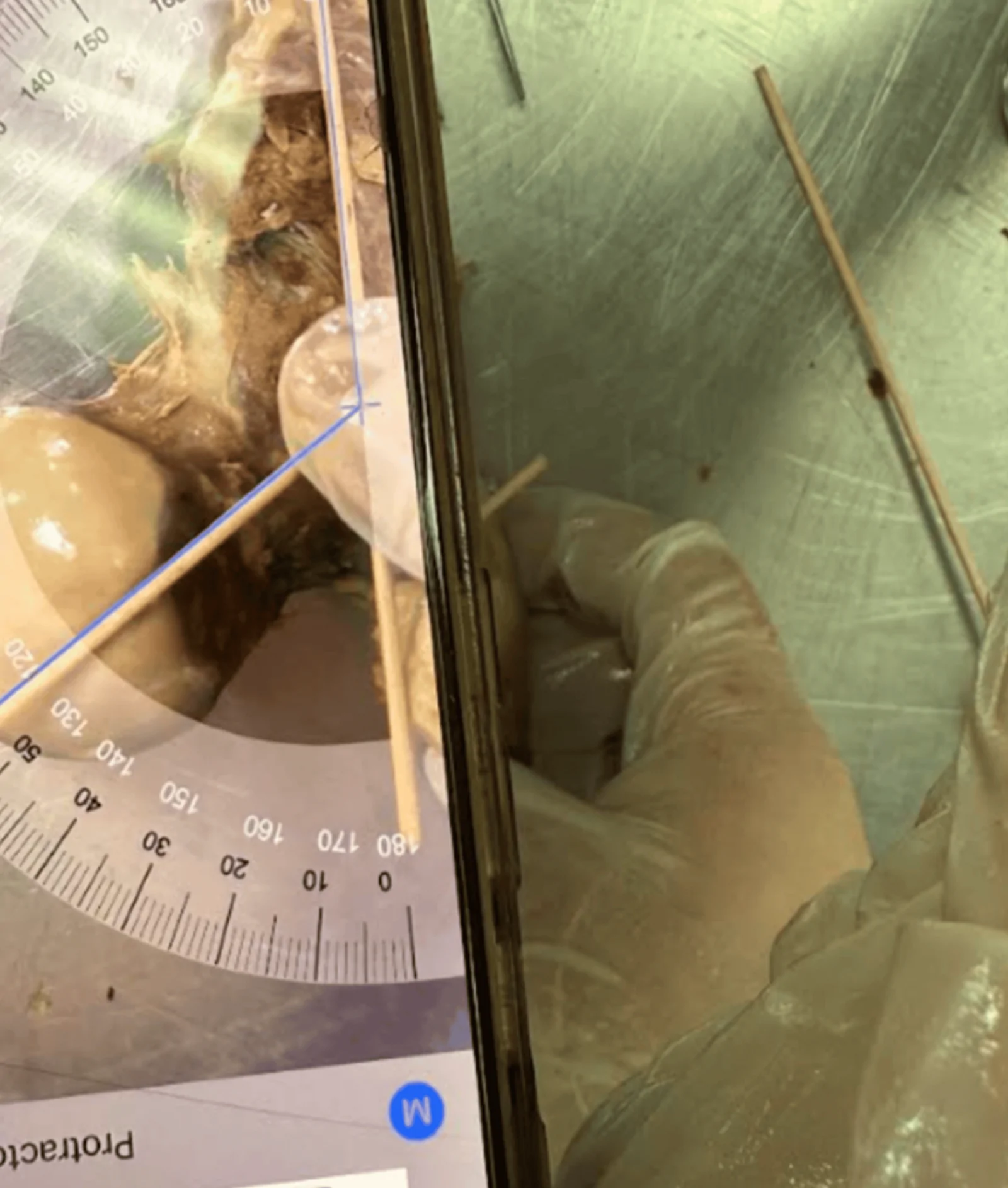
Using protractor application to measure femoral neck shaft angle

Neck-shaft angle (degrees) was measured using the following: stick A was placed along the femoral shaft from the greater to the lesser trochanter; stick B was placed from the center of the femoral head to the midpoint of the intertrochanteric line (Figure 3). The angle between the sticks was recorded using a validated digital protractor application (Figure 4). A physical protractor was used to verify application accuracy.

**Figure 5:**
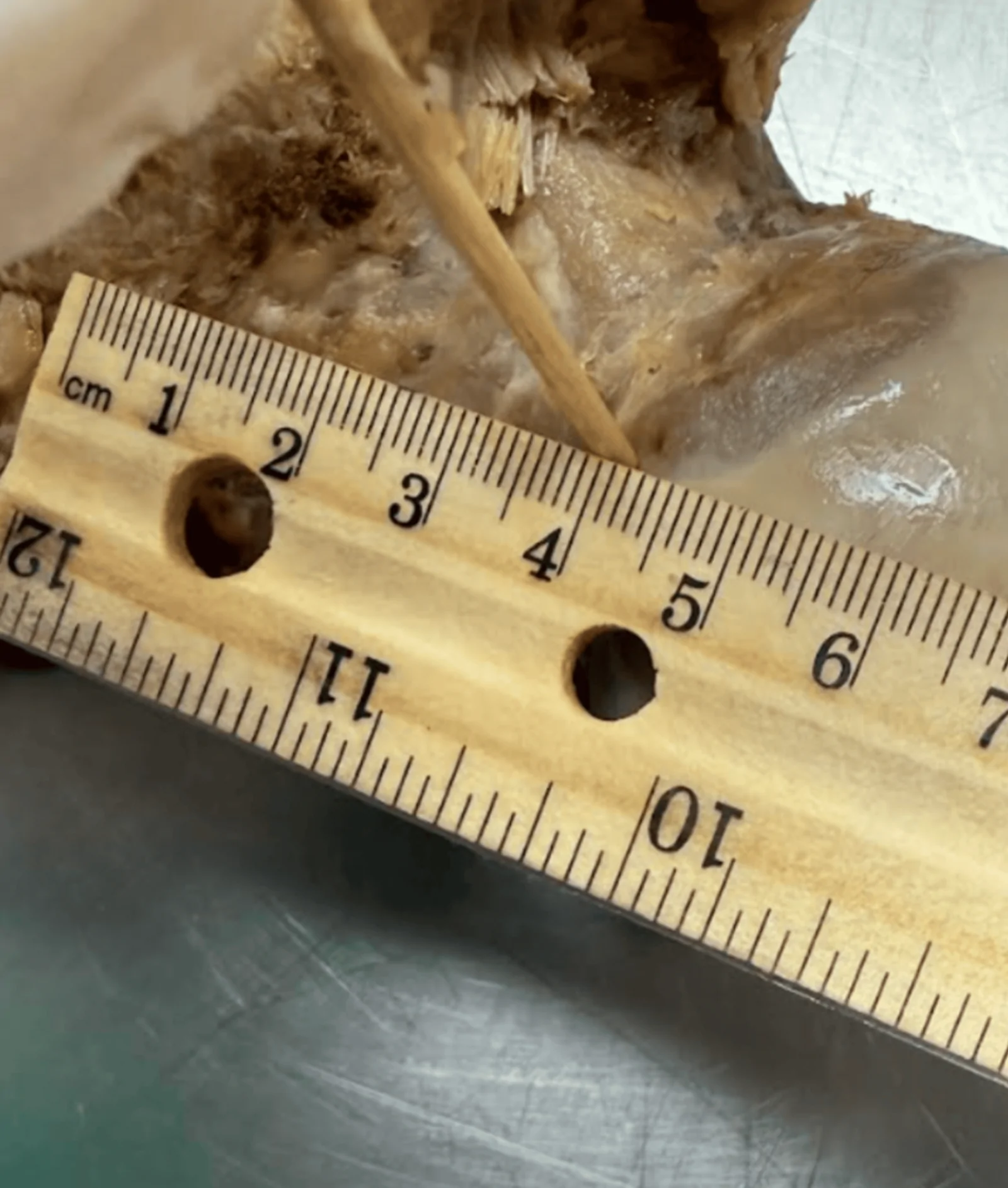
Intertrochanteric line distance to anatomical neck

A straight-edge ruler was used to measure the distance along the neck's long axis from the base of the anatomic neck to the intertrochanteric line (Figure 5).

Statistical analysis

Cadaver-level mean values were used as the primary unit of analysis to avoid pseudoreplication; bilateral values were averaged when both sides were available. For cadavers with unilateral measurements only, the available value was used directly. Analyses were conducted using Microsoft Excel v.16.109.3 (Microsoft, Redmond, Washington) and SPSS Statistics v.31.0.0.0 (IBM Inc., Armonk, New York). Independent-samples Welch t-tests were performed to compare sex-based differences in femoral neck circumference, neck-shaft angle, and femoral neck axis length. Paired-samples t-tests were used to assess right-left differences within cadavers with complete bilateral data (n = 22 paired cadavers). Pearson correlation analysis was conducted to evaluate bilateral symmetry in femoral neck circumference among paired specimens. Shapiro-Wilk tests and Q-Q plot inspection indicated approximate normality for cadaver‑level circumference, angle, and axis‑length distributions, supporting the use of parametric tests. Effect sizes were calculated using Hedges' g to account for small sample size. Statistical significance was defined as α = 0.05. All analyses were performed using standard parametric statistical procedures.

## Results

Descriptive statistics

A total of 47 femora were analyzed (22 right, 25 left). The mean neck-shaft angle measured 127.73° ± 9.08° on the right and 128.16° ± 8.37° on the left. Mean femoral neck axis length measured 4.05 ± 0.50 cm on the right and 4.11 ± 0.53 cm on the left. Mean femoral neck circumference measured 10.13 ± 1.12 cm on the right and 10.12 ± 1.03 cm on the left. No statistically significant side-to-side differences were observed for circumference (paired t = 0.88, p = 0.39), angle (paired t = -0.18, p = 0.86), or neck length (paired t = -0.84, p = 0.41), indicating overall bilateral symmetry across all three parameters. Descriptive statistics by side (Table 1) are reported at the femur level, whereas sex‑based comparisons and bilateral symmetry analyses are conducted at the cadaver level to avoid pseudoreplication. 

**Table 1:** Descriptive statistics and sex comparisons for femoral neck measurements Values are presented as mean (SD). p‑values were calculated using independent‑samples t‑tests comparing males and females within each side. Statistically significant results (p < 0.05) are shown in bold.

Variable	Right, overall (n = 22)	Right, males (n = 10)	Right, females (n = 12)	t (right)	p (right)	Left, overall (n = 25)	Left, males (n = 12)	Left, females (n = 13)	t (left)	p (left)
Circumference, cm	10.13 (1.12)	10.91 (0.76)	9.48 (0.94)	3.95	< 0.001	10.12 (1.03)	10.92 (0.65)	9.38 (0.70)	5.69	< 0.001
Angle, degrees	127.73 (9.08)	127.30 (11.06)	128.08 (7.55)	-0.19	0.85	128.16 (8.38)	125.58 (8.32)	130.54 (8.01)	-1.52	0.14
Neck length, cm	4.05 (0.50)	4.18 (0.55)	3.94 (0.44)	1.11	0.28	4.11 (0.52)	4.33 (0.48)	3.91 (0.49)	2.18	0.04

Sex-based comparisons

Primary analyses used cadaver-level averaged bilateral measurements (or unilateral values when only one side was available) as the unit of analysis.

**Figure 6:**
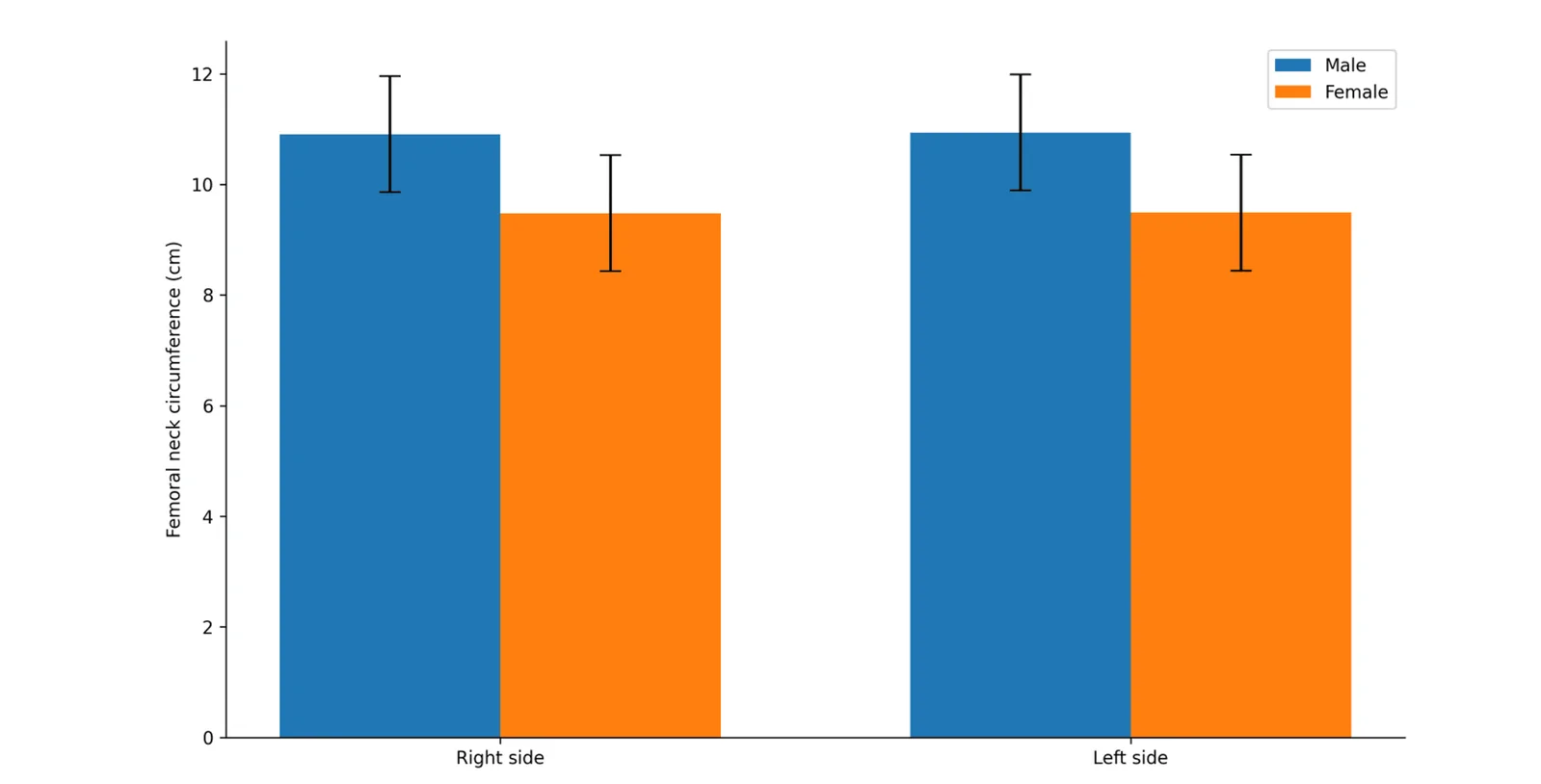
Sex-based differences in femoral neck circumference by side Bars represent mean + standard deviation for right and left femora separately. Right femora included 10 male and 12 female specimens; left femora included 12 male and 13 female specimens.

Male cadavers demonstrated significantly larger mean femoral neck circumference compared to female cadavers (10.93 ± 0.64 cm vs. 9.45 ± 0.77 cm; Welch t(22.74) = 5.22, p < 0.001). The mean difference was 1.47 cm (95% CI: 0.89 to 2.06), with a very large effect size (Hedges' g = 2.00) (Figure 6).

**Figure 7:**
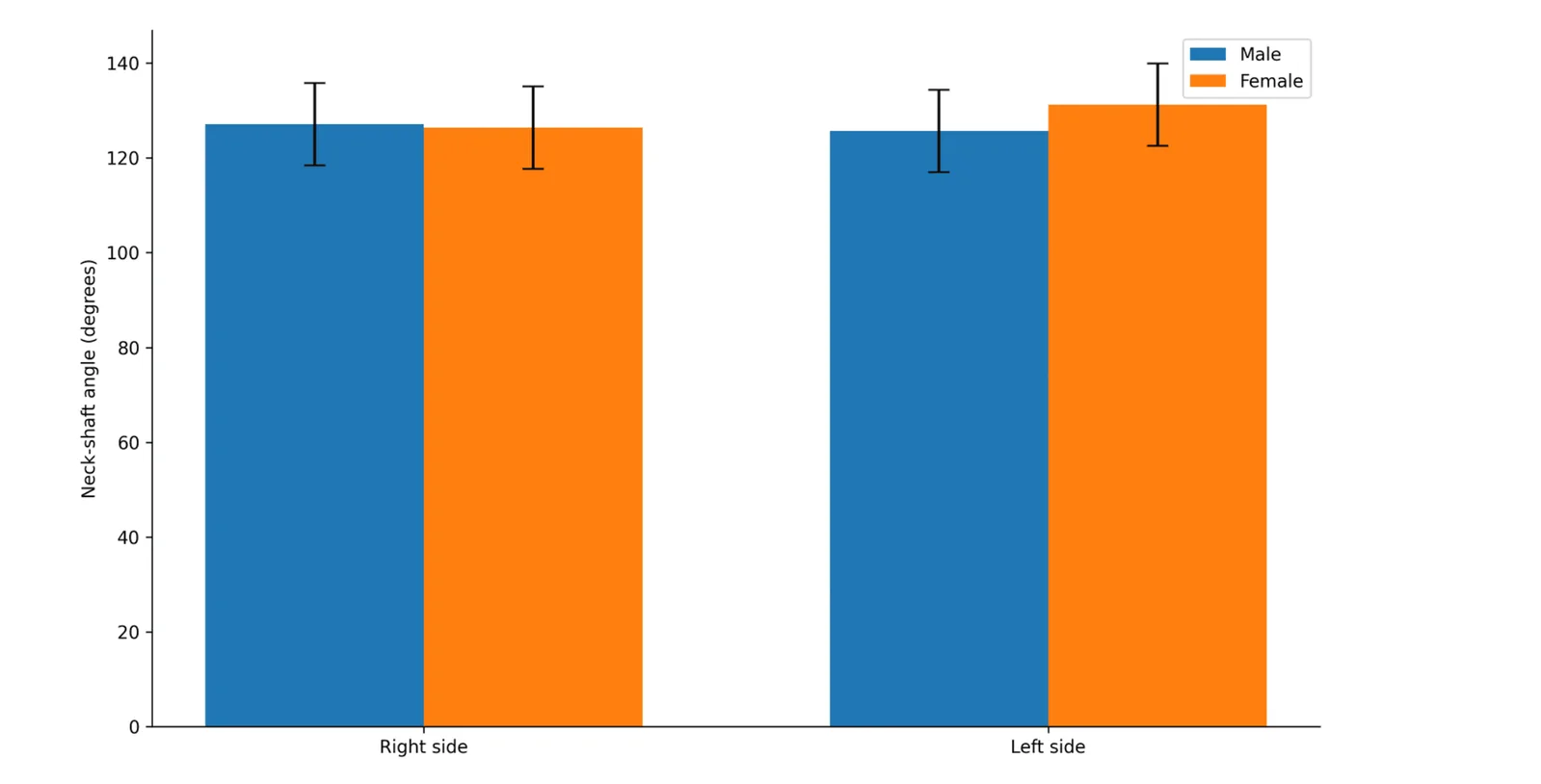
Femoral neck-shaft angle by sex and side Bars represent mean + standard deviation for right and left femora separately. Right femora included 10 male and 12 female specimens; left femora included 12 male and 13 female specimens. Cadaver-level analysis demonstrated no statistically significant sex-based difference in neck-shaft angle (Welch t(20.57) = -0.74, p = 0.47).

**Figure 8:**
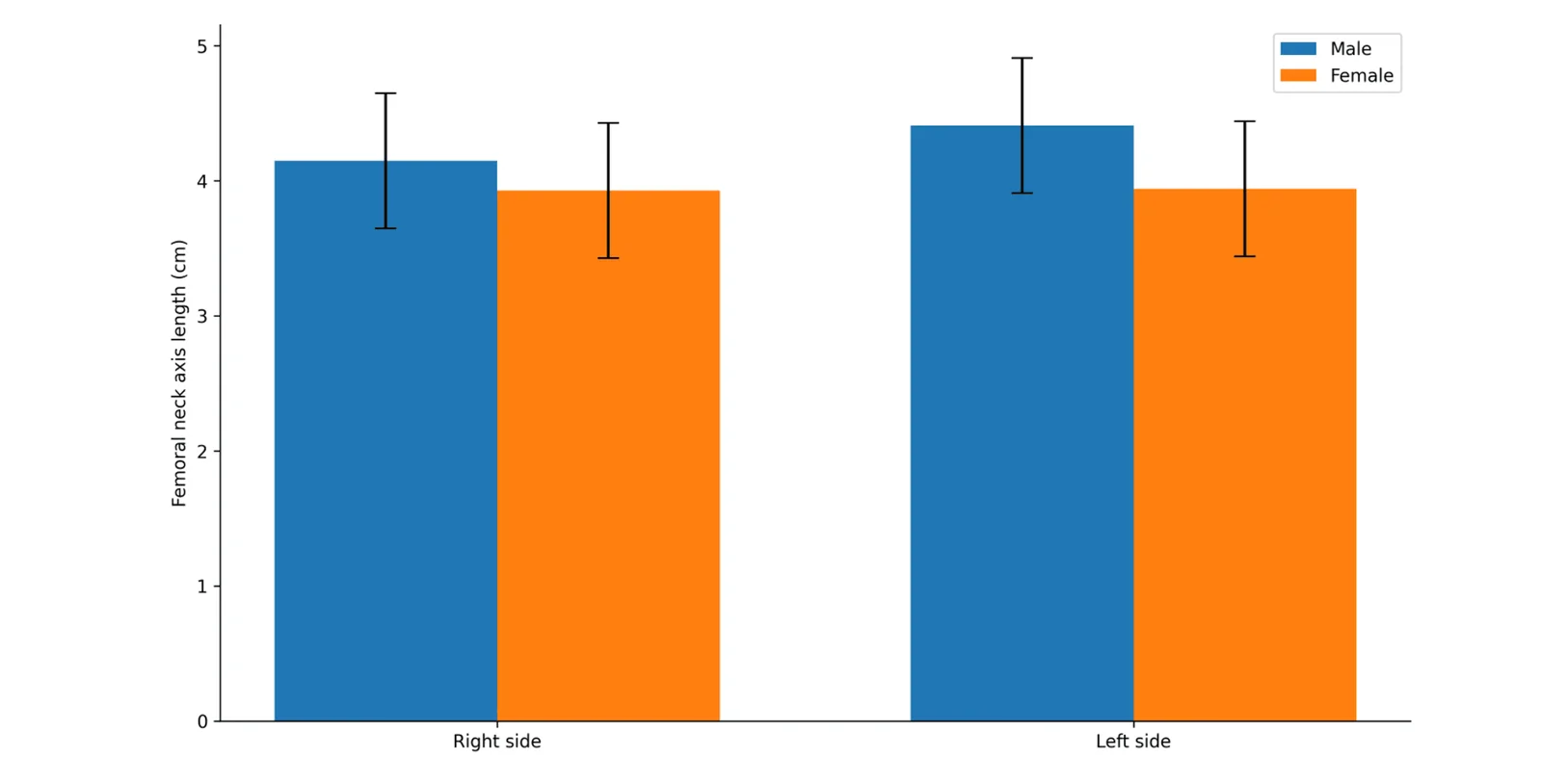
Femoral neck axis length by sex and side Bars represent mean + standard deviation for right and left femora separately. Right femora included 10 male and 12 female specimens; left femora included 12 male and 13 female specimens. Cadaver-level analysis demonstrated no statistically significant sex-based difference in femoral neck axis length (Welch t(22.32) = 1.87, p = 0.074).

No statistically significant sex-based differences were observed in mean neck-shaft angle (males: 126.83° ± 8.63° vs. females: 129.12° ± 6.59°; Welch t(20.57) = −0.74, p = 0.47) (Figure 7) or femoral neck axis length (males: 4.26 ± 0.47 cm vs. females: 3.92 ± 0.43 cm; Welch t(22.32) = 1.87, p = 0.074) (Figure 8).

These findings indicate that femoral neck circumference exhibits pronounced sexual dimorphism, whereas angular and linear measures do not demonstrate statistically significant differences at the cadaver level.

Correlation and bilateral symmetry analysis

**Figure 9:**
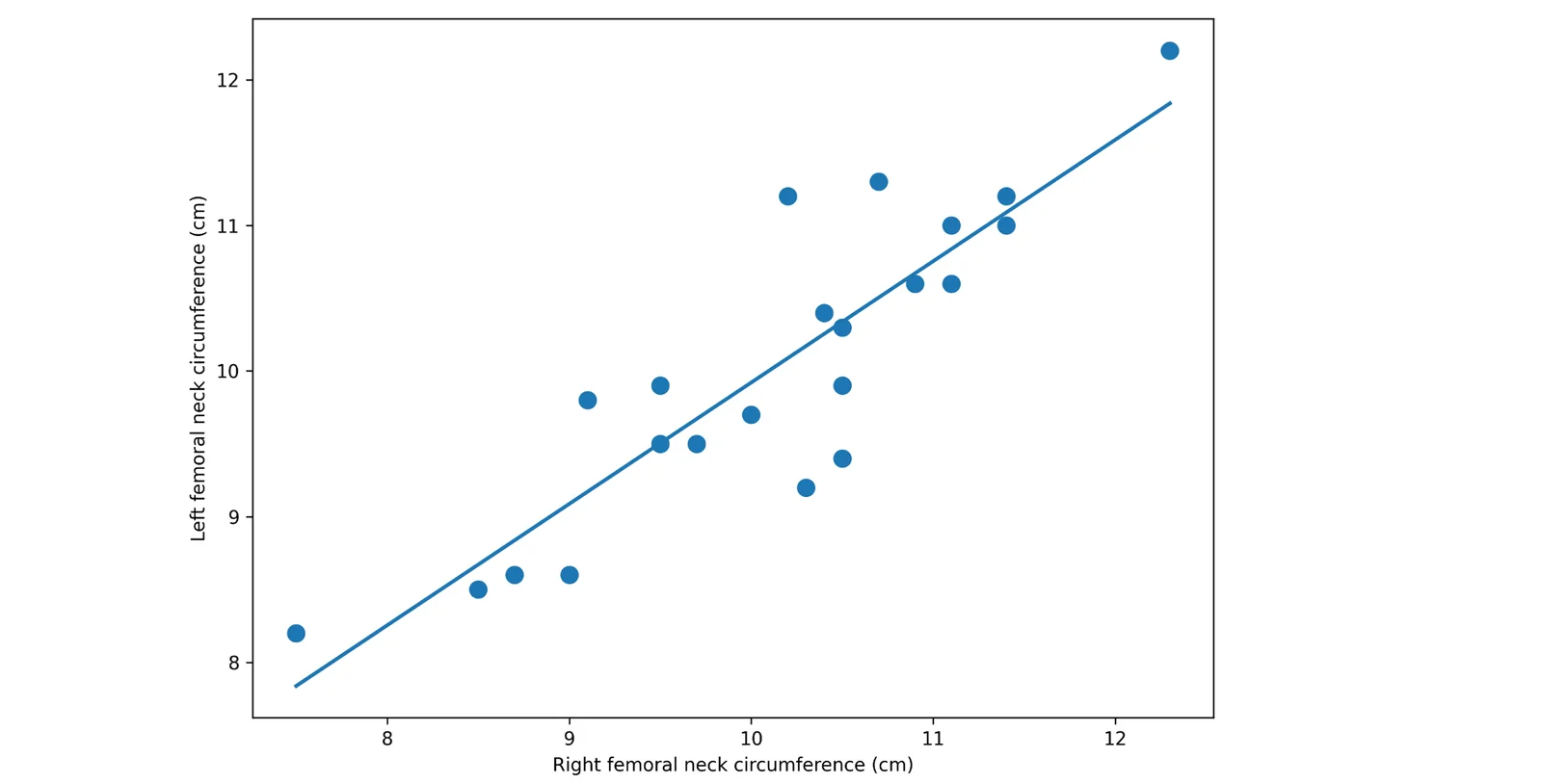
Bilateral symmetry of femoral neck circumference Each point represents one cadaver with complete right and left femoral neck circumference measurements (n = 22 paired cadavers; 44 femora total). Pearson correlation demonstrated strong bilateral symmetry (r = 0.88, p < 0.001). The line represents the least-squares regression fit.

Among cadavers with complete bilateral measurements (n = 22 paired cadavers), femoral neck circumference demonstrated strong bilateral symmetry (Pearson r = 0.88, p < 0.001). Moderate-to-strong bilateral correlations were also observed for neck-shaft angle (r = 0.56, p = 0.007) and femoral neck axis length (r = 0.81, p < 0.001). Paired-samples t-tests confirmed no significant right-left differences for circumference (t(21) = 0.88, p = 0.39), neck-shaft angle (t(21) = -0.18, p = 0.86), or axis length (t(21) = -0.84, p = 0.41) (Figure 9).

## Discussion

This cadaveric study demonstrates clear sexual dimorphism in femoral neck circumference, with males exhibiting substantially larger values (Hedges' g = 2.00). In contrast, neck-shaft angle and femoral neck axis length showed no significant sex differences. These findings suggest that cross-sectional geometry, rather than angular or linear dimensions, represents a primary axis of proximal femoral sexual dimorphism.

These results are consistent with prior imaging-based literature documenting sex-dependent differences in femoral neck structural properties. Duan et al. demonstrated that sexual dimorphism in femoral neck fragility has origins in differential periosteal and endocortical modeling during skeletal growth and aging, with males exhibiting greater displacement of cortex from the femoral neck neutral axis and 13.4% greater bending strength than females in young adulthood [9]. Similarly, Carpenter et al. reported that men had a 46% higher bending strength (My) and 23% higher axial compressive strength (Fy) at the femoral neck after correction for height and weight, with a larger minimum cross-sectional area in males [13]. Beck et al. further documented sex-dependent trends in femoral structural properties across aging cohorts [10]. The current data extend these observations by providing a direct circumferential measurement, which has been largely absent from prior morphometric analyses that relied on radiographic neck width or DXA-derived estimates.

Femoral neck circumference reflects cross-sectional size and is indirectly related to parameters such as cross-sectional area and bending resistance, which contribute to the second moment of inertia [5,6]. Hip fractures in older adults most commonly result from sideways ground-level falls, which generate high bending moments at the superolateral femoral neck [17,18]. Cadaveric fall-loading experiments have confirmed that fracture initiation frequently occurs in this region, with fracture force strongly correlated with femoral neck bone mineral density and geometry [18]. Soft tissue attenuation and fall configuration additionally modulate the internal femoral loading environment, acting as variables independent of bony geometry, as shown in observational video-based evidence [19,20]. 

Notably, the absence of significant sex-based differences in neck-shaft angle stands in partial contrast to findings from Fajar et al.'s meta-analysis, which identified femoral neck angle as a statistically significant risk factor for femoral neck fractures (OR = 1.47; 95% CI: 1.01-2.15) [8]. However, that meta-analysis examined the association between neck-shaft angle and fracture risk - a different question from sex-based dimorphism in angle. The present data suggest that neck-shaft angle may not independently explain the epidemiologic sex disparity in fracture incidence, consistent with prior cadaveric and radiographic studies showing limited or no significant sex differences in neck-shaft angle [8,11]. Furthermore, displaced intracapsular femoral neck fractures (Garden III-IV) carry clinically significant risk for avascular necrosis due to disruption of the retinacular blood supply, potentially necessitating arthroplasty [17,21]. Understanding the geometric substrate of fracture risk is therefore not only of epidemiologic interest but also of direct surgical relevance.

The strong bilateral symmetry in femoral neck circumference (r = 0.88, p < 0.001) observed in this study supports the validity of contralateral morphometric modeling in preoperative planning and implant templating, a clinically practical implication. The absence of strong correlation between circumference and angular or linear variables reinforces the multidimensional nature of proximal femoral geometry and suggests that single-parameter scaling for implant sizing or fracture risk assessment may oversimplify anatomical variability.

Limitations

Several limitations of this study warrant discussion. The sample size was limited to 25 cadavers with 47 total femora, which constrains statistical power for subgroup analyses. Importantly, cadaver demographic data including age, height, weight, BMI, and bone health history were unavailable from institutional records and could not be incorporated into the analysis. Observed sex differences may partially reflect unmeasured differences in body size. The absence of these covariates is a recognized limitation: body size correlates with bone dimensions, and age and BMI influence bone geometry and mineral density. Future studies should prospectively record these variables to enable covariate-adjusted analyses. Because embalmed cadaveric specimens were used, dynamic in vivo responses to loading - such as viscoelastic bone behavior - cannot be directly assessed. Several specimens had radiographic or gross evidence of osteoarthritis, which may influence local femoral neck geometry; these were retained due to the pragmatic cadaveric context but may contribute to unmeasured heterogeneity. The measurement methods used (inextensible filament, digital protractor, ruler) are low-cost and accessible, but future studies should consider validation against CT-derived or three-dimensional morphometric analysis. Additionally, the cadaveric cohort may not be representative of the broader population, and replication in larger, demographically characterized samples across diverse populations is needed.

## Conclusions

This cadaveric morphometric study demonstrates that femoral neck circumference is significantly greater in males than in females, while neck-shaft angle and femoral neck axis length do not differ significantly between sexes. These findings suggest that circumferential cross-sectional geometry may represent a more prominent dimension of proximal femoral sexual dimorphism than angular or linear parameters.
